# Non-Transplantable Recurrence After Initial Liver Resection of Hepatocellular Carcinoma: A Narrative Review

**DOI:** 10.3390/cancers18020317

**Published:** 2026-01-20

**Authors:** Dima Malkawi, Ioannis A. Ziogas, Ana L. Gleisner, Richard D. Schulick, Dimitrios P. Moris

**Affiliations:** 1 Department of Surgery, University of Colorado Anschutz, Medical Campus, Aurora, CO 80045, USA; ioannis.ziogas@cuanschutz.edu (I.A.Z.); ana.gleisner@cuanschutz.edu (A.L.G.); richard.schulick@cuanschutz.edu (R.D.S.); 2 Department of Surgery, MedStar Georgetown Transplant Institute, Washington, DC 20007, USA; dimmoris@yahoo.com; 3 School of Medicine, European University of Cyprus, 1516 Nicosia, Cyprus

**Keywords:** hepatocellular carcinoma, liver resection, liver transplantation, non-transplantable recurrence, risk stratification

## Abstract

**Background/Objectives**: Hepatocellular carcinoma (HCC) constitutes a leading cause of mortality worldwide. Liver transplantation (LT) and liver resection (LR) represent the main curative-intent treatment modalities for early-stage HCC. LT can offer the advantage of both removing the HCC and alleviating the potential underlying liver disease, yet its application is limited by organ scarcity, waitlist dropout, and eligibility criteria. Hence, LR remains widely used due to greater accessibility but is associated with high recurrence rates. Salvage LT is a treatment option for patients with HCC recurrence post-LR, but up to 40% of patients develop non-transplantable recurrence (NTR), defined as recurrence beyond transplant criteria, which precludes LT and is associated in poor outcomes. **Methods**: The present review aims to summarize the current state of evidence on the comparison of LT and LR, the management of recurrent HCC, and the risk factors associated with NTR. **Results**: Clinical and histopathologic factors consistently associated with NTR across studies include larger tumor size, multiple tumors, elevated alpha-fetoprotein levels, underlying liver fibrosis or cirrhosis, microvascular invasion, and satellite nodules—features that reflect aggressive tumor biology and impaired hepatic reserve. **Conclusions**: Improved preoperative risk stratification and identification of patients at high risk for NTR is essential to inform optimal treatment selection.

## Simple Summary

Hepatocellular carcinoma is a form of liver cancer that is associated with high rates of mortality worldwide. The main treatment options for patients with hepatocellular carcinoma include surgical removal of the tumor or undergoing a liver transplant. Although removing the tumor is less invasive than a liver transplant, the cancer frequently recurs. In certain circumstances, the recurrence can be aggressive and not amenable to curative treatments such as liver transplant. Therefore, we aimed to determine what factors put patients at higher risk for this kind of aggressive recurrence to help identify which patients would benefit most from early transplant versus tumor removal alone. A clearer understanding of these risk factors can improve decision-making, reduce the chance of losing the opportunity for a transplant, and guide more personalized care. The findings may also help researchers design better prediction tools and future studies to improve outcomes for people with liver cancer.

## 1. Introduction

Hepatocellular carcinoma (HCC) carries a significant burden globally and ranks as the third leading cause of cancer-related deaths worldwide [1,2]. The incidence of HCC has increased by 75% between 1990 and 2015, in part due to an aging population [3]. Metabolic dysfunction-associated steatotic liver disease (MASLD) and infection by hepatitis B and C viruses are the main risk factors for the development of HCC [4,5]. The implication of cirrhosis in about 80–90% of HCC patients with any underlying liver disease increases the complexity of surgical management for HCC [6]. The Barcelona Clinic Liver Cancer (BCLC) staging system remains the most broadly used framework for the classification and management of HCC as it accounts for tumor burden, liver function, performance status, and cancer-related symptoms [7,8]. According to BCLC, HCC is classified into five stages and the potentially curative modalities for very early (0) and early (A) stage HCC include ablation, liver resection (LR), and liver transplantation (LT) [7].

In an ideal scenario with unrestricted donor organ availability, LT would be the optimal treatment option for HCC as it eliminates both the tumor and the underlying cirrhotic “field effect” [9,10,11]. However, the persistent shortage of donor organs limits the broader application of LT for HCC and necessitates stringent candidate selection. Consequently, LR remains the preferred strategy for patients with HCC in the absence of cirrhosis, as well as for selected patients with cirrhosis but preserved liver function and lack of portal hypertension, provided that tumor size and future liver remnant function are adequate [11]. Nevertheless, HCC recurrence in the liver remnant after LR remains as high as 50–70% in patients with chronic liver disease [12,13]. Although salvage LT has been associated with favorable outcomes for recurrent HCC, it is not feasible for all patients with HCC recurrence as some patients develop non-transplantable recurrence (NTR) despite strict surveillance post-LR [14,15].

The aim of this review is to summarize the current state of evidence on the comparison of LR and LT for the management of HCC, on the treatment of recurrent HCC, and on risk factors of NTR.

## 2. Liver Transplantation Versus Liver Resection

Treatment of early-stage HCC is often conceptualized within a “therapeutic hierarchy,” in which LT and LR provide the greatest survival benefit [16,17]. For appropriately selected patients, upfront or primary LT has been associated with excellent outcomes. Patient selection for LT is primarily guided by the Milan criteria (MC), established by Mazzaferro et al. in 1996 [18], which define transplant eligibility as a single tumor ≤ 5 cm or up to three tumors each ≤3 cm in the absence of macrovascular invasion or extrahepatic spread. Outcomes under these criteria have demonstrated a 5-year survival rate exceeding 70% and recurrence rates of less than 15% [18,19,20]. However, the MC have been criticized for being overly restrictive, prompting some centers to adopt expanded transplantation criteria for patients with HCC. The University of California, San Francisco (UCSF) criteria, proposed in 2001 [21], expanded these limits to include a single tumor ≤ 6.5 cm, up to three tumors each ≤4.5 cm, with a total tumor diameter ≤ 8 cm, achieving survival outcomes comparable to those observed under MC (Table 1). Several other groups subsequently suggested the Milan criteria were too strict and excluded patients with HCC that could benefit from LT, and that the criteria could be further expanded. As a result, Mazzaferro et al. proposed the up-to-seven criteria in which seven was the sum of the size of the largest tumor (in cm) and the number of tumors [22].

However, the use of LT is constrained by the limited donor organ availability, the risk of tumor progression during the waiting period, and eligibility restrictions. Due to these limitations, LR can be a more timely and feasible treatment for HCC. In appropriately selected patients, LR has been associated with a 5-year survival rate of 60–80% but also with a high recurrence risk of 50–70% within 5 years [23,24]. In patients with tumors within the MC, recurrence-free survival has also remained low, ranging from 40 to 48% at 5 years [25,26,27]. A meta-analysis including patients with HCC within the MC showed no difference in overall survival between LR and LT for patients with solitary lesions in studies published after 2010 [28]. Given the lack of data from randomized clinical trials, selecting between LT and LR for eligible patients requires careful risk assessment, considering the risks of post-hepatectomy liver failure, tumor recurrence, as well as local donor availability [11].

## 3. Treatment of Recurrent HCC

Treatment for recurrent HCC is highly individualized, depending on the patient’s performance status, tumor burden, and the location of the recurrent tumor. Recurrence following LR may represent either true metastatic spread from the index tumor or de novo tumorigenesis arising from the diseased liver parenchyma, with important implications for prognosis and treatment response [23,29]. Early recurrence, typically defined as occurring within 6–12 months of resection, is more often associated with aggressive tumor biology and adverse pathological features, whereas late recurrence more commonly reflects multicentric carcinogenesis [13,23]. Repeat LR has been described as a potentially curative approach for intrahepatic HCC recurrence, though reported 5-year survival rates vary widely (22–78%) [30,31,32,33]. However, only a minority of patients meet criteria for repeat resection due to limited hepatic reserve, unfavorable tumor location, or multifocal disease. Locoregional therapies may provide disease control in non-surgical candidates but are rarely curative. To address the challenges of organ scarcity and prolonged waiting times for LT, Majno et al. introduced the concept of salvage LT in 2000. This strategy involves initial LR for patients with a solitary HCC ≤ 5 cm, reserving LT for recurrence or deteriorating liver function, provided the recurrence remains within transplantable criteria [34].

Salvage LT represents the most definitive treatment for recurrent HCC, as it simultaneously addresses tumor recurrence and underlying liver disease. A recent meta-analysis demonstrated that salvage LT provides a survival benefit over repeat LR for patients with recurrent HCC [35]. Furthermore, reported 5-year survival rates following salvage LT for recurrent HCC are also comparable to those of primary LT, ranging between 54 and 80% [36,37,38]. However, the application of salvage LT is restricted to patients whose recurrence falls within established criteria, such as the MC or UCSF. Unfortunately, up to 40% of patients with recurrent HCC present with recurrence outside transplantable criteria, termed NTR [14,15].

### Non-Transplantable Recurrence (NTR)

NTR refers to HCC recurrence following curative-intent treatment that falls outside of established eligibility criteria for LT, such as MC or UCSF criteria, thereby precluding LT as a therapeutic option. Most studies define NTR based on morphologic criteria that mirror commonly used transplant eligibility thresholds. The Milan criteria remain the most frequently applied benchmark, classifying recurrence as non-transplantable when tumor burden exceeds a single lesion greater than 5 cm or more than three lesions each greater than 3 cm, or when macrovascular invasion or extrahepatic disease is present. However, other studies have adopted expanded criteria, including the University of California, San Francisco criteria, or the up-to-seven criteria, reflecting institutional practice patterns and evolving transplant paradigms. As a result, recurrence considered non-transplantable in one study may be classified as transplantable in another, depending on the criteria applied. As anticipated, NTR has been associated with significantly worse outcomes when compared to recurrence within transplant criteria [39]. Therefore, early identification of patients at high risk for NTR at the time of initial presentation is paramount to guiding the optimal primary management—determining whether to proceed with upfront LT or LR for early-stage, transplantable HCC.

## 4. Clinicopathologic Risk Factors for NTR

Several studies have sought to identify predictors of NTR. Both histopathologic and clinical risk factors have been evaluated to refine patient stratification and optimize the selection between LR and LT. A range of clinical and pathological variables have been reported to correlate with an increased risk of NTR; however, the specific factors identified as significant vary among studies (Table 2). Preoperative predictors of NTR include the presence of cirrhosis, multifocal tumors, elevated alpha-fetoprotein (AFP) levels, and larger tumor size. Among patients who have already undergone LR, histopathologic findings such as microvascular invasion and microsatellite lesions have been associated with a higher risk of NTR.

### 4.1. Tumor Size and Number

Increasing tumor size and number of tumors are often indicative of more aggressive tumor biology and are well-recognized risk factors associated with recurrence and survival following both LR and LT for HCC [29,47,48,49,50,51,52,53]. Several studies have demonstrated a significant association between tumor burden and the risk of NTR. Fuks et al. [40] and Zhang C et al. [46] identified HCC size > 3 cm as an independent predictor of NTR, while Pelizzaro et al. [45] and Feng et al. [43] reported a higher probability of NTR in patients with tumors ≥ 4 cm and >5 cm, respectively. In contrast, Gelli et al. [14] and Zhang X et al. [15] did not show a significant association between tumor size and NTR.

Multifocal HCC was also significantly associated with NTR across multiple studies. Lima et al. used the tumor burden score (TBS)—which integrates maximum tumor diameter and number of lesions—as a surrogate measure of tumor load and showed that patients with a high TBS had a significantly greater risk of NTR [44].

### 4.2. Alpha-Fetoprotein (AFP)

Elevated AFP levels also reflect aggressive tumor biology and are associated with negative pathological features, particularly microscopic vascular invasion [6,54]. AFP level has been described as a significant risk factor for NTR though with variability in the cut-off value used in each study. A cut-off AFP level as low >10 ng/mL has been associated with NTR [46], while other studies have reported that AFP levels > 100 ng/mL or >400 ng/mL herald a higher risk of NTR [14,43,45].

### 4.3. Liver Function

Underlying liver fibrosis and cirrhosis has been associated with NTR following LR. The presence of cirrhosis has been linked to intrahepatic metastasis and de novo recurrence of HCC following LR [23,29]. Although other risk factors, such as tumor burden and AFP levels, are readily available pre-operatively, the presence and extent of cirrhosis may not always be apparent preoperatively. Cirrhosis may be present without overt signs, such as portal hypertension, or morphological changes in imaging. Several studies have evaluated liver function using various measures, including the albumin–bilirubin score and assessment of fibrosis, to better predict outcomes after resection. Despite differences in assessment methods, these studies consistently demonstrate that impaired liver function is significantly associated with an increased risk of NTR [14,15,40,42,44].

### 4.4. Pathological Features

Histologic examination of the resected specimen following LR can provide prognostic value and guide subsequent management. Adverse histopathological characteristics including microvascular invasion and microsatellite lesions were shown to be independently associated with NTR across the literature [39,40,41,43,45].

### 4.5. Other Factors Implicated with NTR

In patients undergoing curative intent LR for HCC, anatomical resection (AR) was associated with a significantly lower risk of NTR when compared with non-anatomical resection (NAR) (3-year NTR 9.8% vs. 14.4%) [55]. In a subgroup analysis stratified by TBS, patients with a medium TBS demonstrated a lower risk of NTR following AR compared to NAR (3-year NTR 10.9% vs. 19.1%). In low TBS patients, the type of hepatic resection was not associated with NTR (3-year NTR 8.6% vs. 10.2%) [55].

## 5. Discussion

Although LT may lead to improved recurrence-free survival for many patients with early-stage HCC, its clinical utility is limited by the donor organ scarcity, tumor progression during the waiting period, and subsequent waitlist dropout. LR represents a feasible alternative to LT for selected patients; however, it is associated with high recurrence rates, and a subset of patients develops aggressive recurrence that is not amenable to curative therapy.

The concept of NTR introduces an important clinical distinction: not all recurrences carry the same prognostic implications. Patients who recur within transplantable criteria remain candidates for salvage LT, achieving long-term survival comparable to those who undergo primary LT. In contrast, patients who develop NTR have fewer available treatment options and substantially reduced survival. Early identification of patients at increased risk of NTR is therefore pivotal for optimizing treatment strategy. Specifically, upfront LT may be preferable in patients with a high predicted risk of NTR, whereas LR remains appropriate for low-risk patients who may still benefit from salvage LT if recurrence remains within transplant criteria.

The findings summarized in this review highlight that NTR represents a clinically meaningful endpoint following LR for HCC. While recurrence after liver resection (LR) has long been acknowledged as common, the differentiation between transplantable and non-transplantable recurrence highlights that not all recurrences carry equivalent prognostic or therapeutic consequences. In patients with transplantable recurrence, the opportunity for salvage LT is preserved, whereas patients with NTR face therapeutic limitations and markedly worse survival outcomes [14,15,39]. This distinction underscores the need for refined risk stratification strategies at the time of initial treatment selection. As such, identifying patients at high risk for NTR should be a central objective in work up and treatment planning.

Several studies have proposed risk factors to guide the selection between LT and LR; however, there remains no consensus on their incorporation into clinical decision-making. The studies summarized in this review provide a framework to stratify the risk of NTR. A recurring theme across studies is that NTR is driven by markers of aggressive tumor biology rather than technical aspects of surgery alone. Risk factors consistently associated with NTR among several studies include tumor size > 3 cm, multifocal disease, cirrhosis or advanced fibrosis, and high AFP levels, with additional contributions from vascular invasion, satellite nodules, and high TBS. Together, these factors reflect aggressive tumor biology and impaired hepatic reserve, both of which predispose to recurrence patterns that exceed transplant criteria. Importantly, factors such as tumor size and number, liver function, and AFP levels are measurable preoperatively, making them suitable candidates for early risk stratification. Emerging approaches, such as imaging markers (e.g., LI-RADS criteria), systemic inflammatory indices (e.g., neutrophil–lymphocyte ratio), and artificial intelligence-based prediction models offer additional promise in refining preoperative risk assessment. Nonetheless, variability in model accuracy and limited external validation currently restrict their widespread clinical application.

These observations challenge traditional paradigms that prioritize anatomic resectability and liver function while underweighting oncologic risk. Although LR may be technically feasible, it may be oncologically suboptimal in patients with high-risk disease who are unlikely to retain transplant eligibility at recurrence. In such cases, upfront LT may offer superior long-term benefit despite greater initial resource utilization. Traditional selection between LR and LT has relied heavily on morphologic criteria, including tumor size and number, as described by MC, UCSF criteria, and up-to-seven criteria [18,21,22]. While these frameworks have been instrumental in standardizing transplant eligibility and improving outcomes, they incompletely capture tumor biology. Increasing evidence suggests that biologic aggressiveness, rather than tumor burden alone, drives early recurrence and progression beyond transplantable thresholds [9,10,14].

Alpha-fetoprotein (AFP) remains the most widely used surrogate marker of tumor biology and has consistently been associated with recurrence risk and adverse histopathologic features such as microvascular invasion [54]. Multiple studies included in this review demonstrate a stepwise increase in the risk of NTR with rising AFP levels, though the optimal cutoff remains variable across cohorts [14,43,45,46]. Importantly, AFP is readily available preoperatively and can be longitudinally monitored, making it a practical component of risk stratification. The integration of AFP into composite scores, such as the AFP score and tumor burden score (TBS), further enhances predictive accuracy and reflects a shift toward biologically informed selection criteria [14,44,45].

Beyond AFP, emerging markers of tumor biology—including radiologic features (e.g., LI-RADS characteristics), metabolic imaging, and inflammatory indices—have shown promise in predicting aggressive recurrence patterns [46]. Several other markers and imaging studies for diagnosing recurrent HCC have been described, including prothrombin induced by vitamin K absence-II (PIVKA-II), and positron emission tomography (PET), but their role in elucidating NTR is yet to be determined [56,57,58]. While these tools remain heterogeneous and incompletely validated, they represent an important direction for future research. Their incorporation into multidisciplinary evaluation may allow clinicians to identify patients at high risk for NTR who would otherwise appear favorable based on conventional staging alone.

Underlying liver disease plays a dual role in HCC recurrence, contributing both to metastatic spread of the index tumor and to de novo tumor formation within a carcinogenic hepatic microenvironment [6,23,29]. Advanced fibrosis and cirrhosis impair hepatic reserve, limit the feasibility of repeat curative therapies, and are independently associated with NTR across multiple studies [14,15,40,42,44]. The albumin–bilirubin (ALBI) score and fibrosis stage have emerged as objective measures of liver function that correlate with oncologic outcomes following LR. Studies demonstrate that patients with impaired liver function are not only more susceptible to recurrence but also less likely to remain within transplant criteria at the time of recurrence [14,15,40,42,44]. These findings reinforce the concept that patients with borderline liver function and early-stage HCC may derive greater long-term benefit from upfront LT rather than LR, even when tumors fall within conventional transplant criteria.

A key limitation of current risk stratification strategies is that several of the strongest predictors of NTR, such as microvascular invasion and satellite nodules, are only detectable on postoperative histopathology [39,40,41,43,45]. Their presence reflects biologically aggressive disease with a propensity for early dissemination and rapid progression. Therefore, these features have limited utility in preoperative decision-making but may guide subsequent treatment strategies, including sequential LT. Sequential LT, also referred to as pre-emptive LT, de principe LT, or ab initio LT, has been proposed as a proactive approach to reduce HCC recurrence prophylactically [40,59,60,61]. A prospective validation of sequential LT from Barcelona compared sequential with salvage LT on an intention-to-treat analysis and demonstrated favorable outcomes with the sequential strategy; however, the authors proposed to wait at least 6 months before listing since early (<6 months) recurrence reflects aggressive tumor biology leading to tumor extent exceeding transplant criteria [62]. Another comparative study by Tribillon et al. on intention-to-treat and per-protocol analyses showed a statistically significant superior survival rate in the sequential LT group (84.6%) compared to salvage LT (74.8%) [60]. However, implementing sequential LT risks overtreating patients who would not have developed aggressive recurrence, potentially exacerbating the shortage of donor organs. Accurate identification of patients at high risk of NTR is therefore essential to better balance oncologic benefit with organ stewardship—supporting the use of sequential LT in patients with high-risk disease, while reserving repeat LR or salvage LT for those with more favorable tumor biology.

Therefore, based on the current state of evidence, we believe that a multidisciplinary approach that integrates tumor burden, tumor biology, and liver function is warranted for the personalized management of HCC given its heterogeneity. Patients with low-risk features—such as solitary tumors ≤3 cm, low AFP, preserved liver function, and absence of radiologic high-risk features—may reasonably undergo LR with close surveillance and the option of salvage LT. Conversely, patients with high-risk profiles may derive greater benefit from upfront LT or enrollment in protocols evaluating sequential transplantation.

**Figure 1:**
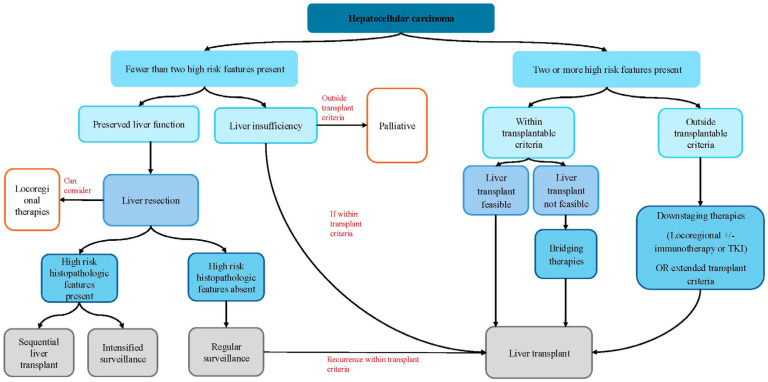
Proposed decision-making algorithm for management of HCC to minimize the risk of non-transplantable recurrence based on pre-operative risk factors.

We proposed a clinical decision-making algorithm for the initial management of HCC summarized in Figure 1. This approach classifies patients with HCC into low-risk or high-risk for NTR (Table 3) which dictates what treatment options may be the most beneficial. The proposed algorithm provides a pragmatic framework to guide multidisciplinary discussions. While these recommendations are not prescriptive, they offer a structured approach to balancing oncologic benefit, surgical risk, and resource allocation.

### Limitations of Current Evidence

Despite the growing body of evidence on NTR, several limitations warrant consideration. The definition of NTR varies across studies, with some defining it as recurrence beyond the MC and others using expanded thresholds such as the UCSF or up-to-seven criteria or including extrahepatic or macrovascular spread. This heterogeneity complicates between-study comparisons and the generalizability of reported outcomes. An additional and increasingly relevant limitation in the interpretation of NTR arises from geographic and practice-related variability in transplant strategies. Most proposed transplant criteria for HCC were developed in the context of deceased donor liver transplantation (DDLT), where organ scarcity necessitates strict selection criteria thresholds. Over the past decade, however, living donor liver transplantation (LDLT) has emerged as a clinically safe alternative to DDLT and now makes up the majority of LT cases in Asian countries [63,64,65]. Because grafts from living donors are not restricted by the organ allocation systems, many centers have adopted expanded selection criteria for LDLT [66]. As a result, recurrence patterns that would be classified as non-transplantable in deceased donor settings may remain transplantable in living donor programs. This practice heterogeneity introduces an additional limitation to the interpretation of NTR across studies, as the clinical relevance of NTR is highly dependent on local transplant policies and donor availability. Moreover, outcomes following LDLT for advanced or biologically aggressive HCC may differ from those observed in DDLT, further confounding interpretation.

Surveillance practices following LR vary widely across institutions and may influence both the detection and classification of recurrence. Feng et al. demonstrated that irregular or absent surveillance was independently associated with a higher risk of recurrence beyond Milan criteria [43]. Delayed detection may allow tumors to progress from potentially transplantable to non-transplantable stages, artificially inflating NTR rates. These findings suggest that surveillance intensity is not merely a passive monitoring strategy but an active determinant of oncologic outcomes. High-risk patients may benefit from more frequent imaging and AFP monitoring to facilitate early detection of recurrence and timely referral for salvage LT. Personalized surveillance protocols tailored to preoperative risk profiles represent an underexplored but potentially impactful strategy to reduce NTR incidence.

These variations in definition have important implications for interpreting recurrence outcomes and identifying risk factors for NTR. Studies conducted in deceased donor-dominant regions may overestimate the clinical finality of certain recurrence patterns when applied to living donor programs. Conversely, expanded transplant criteria may attenuate the apparent impact of NTR while potentially increasing the risk of post-transplant recurrence. Therefore, NTR should be interpreted not as a fixed oncologic outcome but as a functional endpoint reflecting the intersection of tumor biology, recurrence pattern, and transplant feasibility within a given healthcare system.

Most studies examining factors predictive of NTR are retrospective and single-center in design, which increases the risk of selection bias and reduces the strength of available evidence. Patient selection for liver resection or transplantation is often influenced by institutional expertise, local transplant availability, surgeon preference, and evolving practice patterns, all of which may confound observed associations between clinicopathologic factors and recurrence outcomes. Furthermore, retrospective studies are subject to incomplete data capture, variability in surveillance protocols, and inconsistent definitions of recurrence, which may further distort risk estimates and limit reproducibility across centers.

Another important limitation is the relative underrepresentation of etiology-specific analysis. Hepatocellular carcinoma arises from diverse underlying liver diseases, and growing evidence suggests that tumor biology, recurrence patterns, and response to treatment differ according to disease etiology. Etiology-specific variability remains largely underexplored; risk models developed in predominantly hepatitis B virus-endemic populations may not be directly applicable to Western cohorts characterized by higher rates of MASLD or alcohol-related liver disease. Differences in background liver inflammation, fibrosis progression, immune microenvironment, and competing risks of liver-related mortality may substantially alter recurrence dynamics and transplant candidacy in these populations.

Finally, while predictive models offer useful risk stratification, few have been prospectively implemented in clinical decision-making or demonstrated a survival benefit when used to guide the decision between upfront LT versus LR. There is a paucity of prospective studies demonstrating that the use of these models to guide the choice between upfront liver transplantation and liver resection leads to improved survival, reduced recurrence, or more efficient organ utilization. Without prospective validation and integration into multidisciplinary treatment algorithms, the clinical utility of these models remains largely theoretical. Future studies should focus on prospective, multi-center validation across diverse patient populations and assess whether risk-adapted treatment strategies based on predicted non-transplantable recurrence can meaningfully improve oncologic outcomes while preserving responsible stewardship of donor organs.

## 6. Conclusions

NTR represents the most clinically consequential form of recurrence after LR for HCC, as it precludes salvage LT and is associated with poor survival outcomes. Accurate risk stratification of patients with HCC for NTR at the time of initial diagnosis is therefore critical to optimizing treatment selection. While postoperative histopathologic factors provide strong prognostic information, they are not available preoperatively and thus have limited utility in upfront treatment planning. In contrast, preoperative variables, such as tumor size and number, AFP level, and liver function indicators, offer more practical tools for risk stratification. The integration of predictive models into multidisciplinary decision-making may enable more personalized therapy by distinguishing patients who would benefit from upfront LT from those who may safely undergo LR with the option of salvage LT. Future prospective, multi-center studies incorporating molecular biomarkers, radiomics, and artificial intelligence-based prediction tools will be crucial to refine risk stratification and improve survival in patients with early-stage, transplant-eligible HCC.

## Figures and Tables

**Table 1:** Criteria used to assess transplant eligibility in patients with HCC and associated outcomes.

	Criteria	Outcomes
Milan (1996) [18]	Single tumor ≤ 5 cm, OR 2–3 tumors, none exceeding 3 cm AND No vascular invasions and/or extrahepatic spread	4-year actuarial survival: 75% 4-year recurrence-free survival: 83%
UCSF (2001) [21]	Single tumor ≤ 6.5 cm, OR 2–3 lesions, none exceeding 4.5 cm, with total tumor diameter ≤ 8 cm AND No vascular invasions and/or extrahepatic spread	5-year overall survival: 75.2%
Up-to-seven (2009) [22]	Seven as the sum of the size of the largest lesion (in cm) and the number of lesions	5-year overall survival: 71.2%

Abbreviations: UCSF = University of California, San Francisco.

**Table 2:** Risk factors associated with non-transplantable recurrence (NTR) after liver resection for HCC.

Study	Year	Design	N	Definition of NTR	Recurrence (*n*)	NTR (*n*)	Risk Factors
Fuks et al. [40]	2012	Retrospective	112	Recurrence beyond MC	90	30	Microscopic vascular invasion satellite nodules tumor size > 3 cm poorly differentiated tumors liver cirrhosis
Lee et al. [41]	2014	Retrospective	295	Recurrence beyond MC	183	111	Initial disease beyond MC, lymphovascular invasion, microsatellites, presence of multiple tumors
Seo et al. [39]	2020	Retrospective	468	Recurrence beyond MC	211	36	Presence of satellite nodules, microvascular invasion, and unfavorable gross findings (multinodular confluent and infiltrative)
Gelli et al. [14]	2020	Retrospective	148	AFP score > 2, macroscopic vascular invasion, extra hepatic disease or early recurrence (<6 months)	81	31	>1 nodule, AFP > 100 ng/mL, F4 fibrosis
Zhang X et al. [15]	2022	Retrospective	MC: 293 UCSF: 320	Recurrence beyond MC or UCSF criteria	MC: 113 UCSF: 131	MC: 32 UCSF: 35	MC: Tumor size > 3 cm, multiple lesions, F4 fibrosis, portal hypertension UCSF: Tumor size > 3 cm, multiple lesions, F4 fibrosis
Li et al. [42]	2022	Retrospective	309	Recurrence beyond MC	94	35	Cirrhosis, multiple lesions
Feng et al. [43]	2022	Retrospective	753	Recurrence beyond MC	366	138	AFP > 400 ng/mL, tumor size > 5 cm, multiple tumors, microvascular invasion, no/irregular recurrence surveillance
Lima et al. [44]	2023	Retrospective	1620	Recurrence beyond MC	842	341	AFP > 400 ng/mL, high TBS, medium ALBI score
Pelizzaro et al. [45]	2023	Retrospective	512	Recurrence beyond MC or up-to-seven criteria	286	MC: 155 Up-to-seven: 135	MC and up-to-seven: initial tumor size ≥ 4 cm, elevated AFP (>19), microvascular invasion, microsatellite lesions
Zhang C et al. [46]	2024	Retrospective	253	Recurrence beyond UCSF criteria	86	34	LI-RADS features: APHE, washout, capsule, AFP > 10 ng/mL

Abbreviations: AFP = alpha-fetoprotein; ALBI = albumin–bilirubin; APHE = arterial phase hyperenhancement; LI-RADS = Liver Imaging Reporting and Data System; MC = Milan criteria; TBS = tumor burden score; and UCSF = University of California, San Francisco.

**Table 3:** Summary of high-risk clinical and histopathological features for non-transplantable HCC recurrence.

**High-Risk Clinical Features**
Tumor size > 3 cm Multifocal disease (≥2 lesions) Elevated AFP (>100–400 ng/mL) Radiologic evidence of vascular invasion (e.g., washout, capsule, irregular margins) Presence of cirrhosis or advanced fibrosis (F4, elevated ALBI score) High tumor burden score (TBS > 3)
**High-Risk Histopathological Features**
Microvascular invasion Satellite nodules

Abbreviations: AFP = alpha-fetoprotein; ALBI = albumin–bilirubin; and TBS = tumor burden score.

## Data Availability

The original contributions presented in this study are included in the article. Further inquiries can be directed to the corresponding author.

## Abbreviations

The following abbreviations are used in this manuscript:

HCC	Hepatocellular carcinoma
LT	Liver transplant
LR	Liver resection
MASLD	Metabolic dysfunction-associated-steatotic liver disease
BCLC	Barcelona Clinic Liver Cancer
NTR	Non-transplantable recurrence
MC	Milan Criteria
UCSF	University of California, San Francisco
AFP	Alpha-fetoprotein
TBS	Tumor burden score
ALBI	Albumin–bilirubin
APHE	Arterial phase hyperenhancement
LI-RADS	Liver Imaging Reporting and Data System
